# Harnessing artificial intelligence for enhanced public health surveillance: a narrative review

**DOI:** 10.3389/fpubh.2025.1601151

**Published:** 2025-07-30

**Authors:** Vanessa I. S. Mendes, Beatriz M. F. Mendes, Rui Pedro Moura, Inês M. Lourenço, Mariana F. A. Oliveira, Kim Lee Ng, Cátia S. Pinto

**Affiliations:** ^1^Global Digital Health and International Affairs Unit, SPMS - Shared Services of the Ministry of Health, E. P. E., Lisbon, Portugal; ^2^Department of Sequencing and Bioinformatics, Statens Serum Institute, Copenhagen, Denmark

**Keywords:** public health, artificial intelligence, epidemiology, surveillance, medical countermeasures, health threats, early detection, social media data

## Abstract

Artificial intelligence (AI) has a transformative potential to revolutionize public health by addressing critical challenges in disease prevention, outbreak detection, and countermeasures distribution. Traditional public health surveillance methods often face limitations, such as delays in reporting, under-detection of cases, and the overwhelming complexity of managing large datasets. In contrast, AI technologies enable real-time analysis, enhance scalability, and support more effective decision-making, especially during health crises. This review examines the profound impact of AI on key areas of public health, with a particular focus on communicable diseases. It explores how AI-driven technologies are transforming disease monitoring and surveillance, outbreak prevention, and disease modeling, improving the ability to detect and respond to emerging health threats. Furthermore, the role of internet and social media in managing disease outbreaks through AI-powered systems is also highlighted, showcasing how AI can harness information from diverse data sources to enhance public health interventions. The review also delves into the regulatory landscape, emphasizing the importance of robust standards and frameworks, such as those established by the EU, for ensuring the safe, ethical, and responsible implementation of AI in public health. By shedding light on AI’s potential to improve real-time decision-making and support health crisis management, this paper underscores its transformative role in shaping the future of public health surveillance and response.

## Graphical abstract

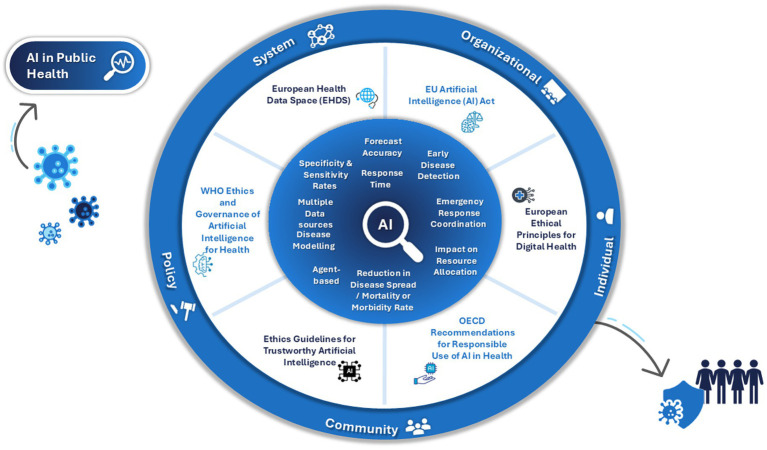

## 1 Introduction

Public health is a multidisciplinary field dedicated to improving the health and well-being of populations through disease prevention, health promotion, and the mitigation of health risks in a community-wide regard. It plays a crucial role in ensuring societal resilience and equity, addressing challenges such as infectious disease outbreaks and the mitigation of its spread, chronic diseases management, and environmental health risks. The preservation of public health relies on strategies that identify health threats, monitor health trends, and implement timely interventions to minimize morbidity and mortality (1, 2).

Traditionally, public health surveillance has relied on manual methods and epidemiological models, built on mathematical models in epidemiology (3). These approaches depend on the systematic collection and analysis of data from healthcare facilities, laboratory testing, and case reporting systems, among other sources. While effective in many contexts, these methods face challenges such as delayed reporting, under-detection of cases, and limited scalability. Additionally, manual methods can struggle to process the vast and rapidly growing volumes of data generated by modern society, particularly with the advent of digital and interconnected technologies, and are not able to adapt, rapidly, to confounding variables and external variables that do not behave in a predictable, linear way (4).

Artificial intelligence (AI) offers transformative potential in addressing these limitations by augmenting traditional public health surveillance methods (5). AI encompasses a range of computational techniques, including machine learning, natural language processing, computer vision, and deep learning, which are designed to analyze, predict, and automate tasks traditionally performed by humans (6). Machine learning, a subset of AI, enables systems to identify patterns in data and make predictions, while natural language processing allows for the analysis of unstructured textual information from diverse sources. These AI tools have already shown remarkable potential in fields such as disease detection, risk prediction, and outbreak modeling (7).

AI’s application in public health has grown significantly in recent years, accelerated by the emergence of both generative AI and the need to give a timely response to the COVID-19 pandemic (8). These studies have focused on addressing areas such as disease surveillance, outbreak detection, predictive modeling, and response assessment. By integrating diverse datasets from healthcare records, social media platforms, environmental conditions, and genomic data, AI has the potential to enable real-time analysis and timely responses to emerging health threats (9).

The goal of this review is to examine the impact of AI in areas related to public health surveillance, particularly for communicable diseases. The analysis seeks to explore the potential of AI-driven technologies to reshape public health practices by improving the speed, scale, and precision of surveillance and response mechanisms. Additionally, the paper addresses key standards and regulations, placing particular emphasis on European Union (EU)-level frameworks, which play a crucial role in promoting the responsible and effective use of AI in public health.

## 2 Methods

The studies were identified based on a literature review covering the period from January 2017 to December 2024. The databases used were PubMed/MEDLINE, Science Direct, IEEE Xplore, and Google Scholar. The following key terms were used to conduct a comprehensive search: “public health,” “artificial intelligence,” “machine learning,” “natural language processing,” “surveillance,” “epidemiology,” “disease modelling,” “deep learning” and “social media.” The review focused on peer-reviewed publications, with additional insights from major public health reports from regulators and world experts. Additional databases, such as EUR-Lex, organization for Economic Co-operation and Development (OCDE), World Health Organization (WHO) and the official webpages of the European Commission, were reviewed to identify relevant standards and EU legislation related to AI and public health. The manuscripts were selected based on the clarity of AI model development, information regarding the training datasets and peer-reviewed validation of their outputs, clarity regarding the model functionalities and applicability to real world scenarios. Out of a pool of candidate publications, a preliminary screening process that combined AI and public health (excluding surgery procedures, robotics, meta-analysis studies, telemedicine, disease type and impact to public health) resulted in 64 viable publications that were used in the manuscript, with 36 as use cases to demonstrate the applicability of AI in public health within the target timeline. These use cases were summarized in Supplementary Table S1.

## 3 Results

### 3.1 Discussion: AI and public health surveillance

**Figure 1:**
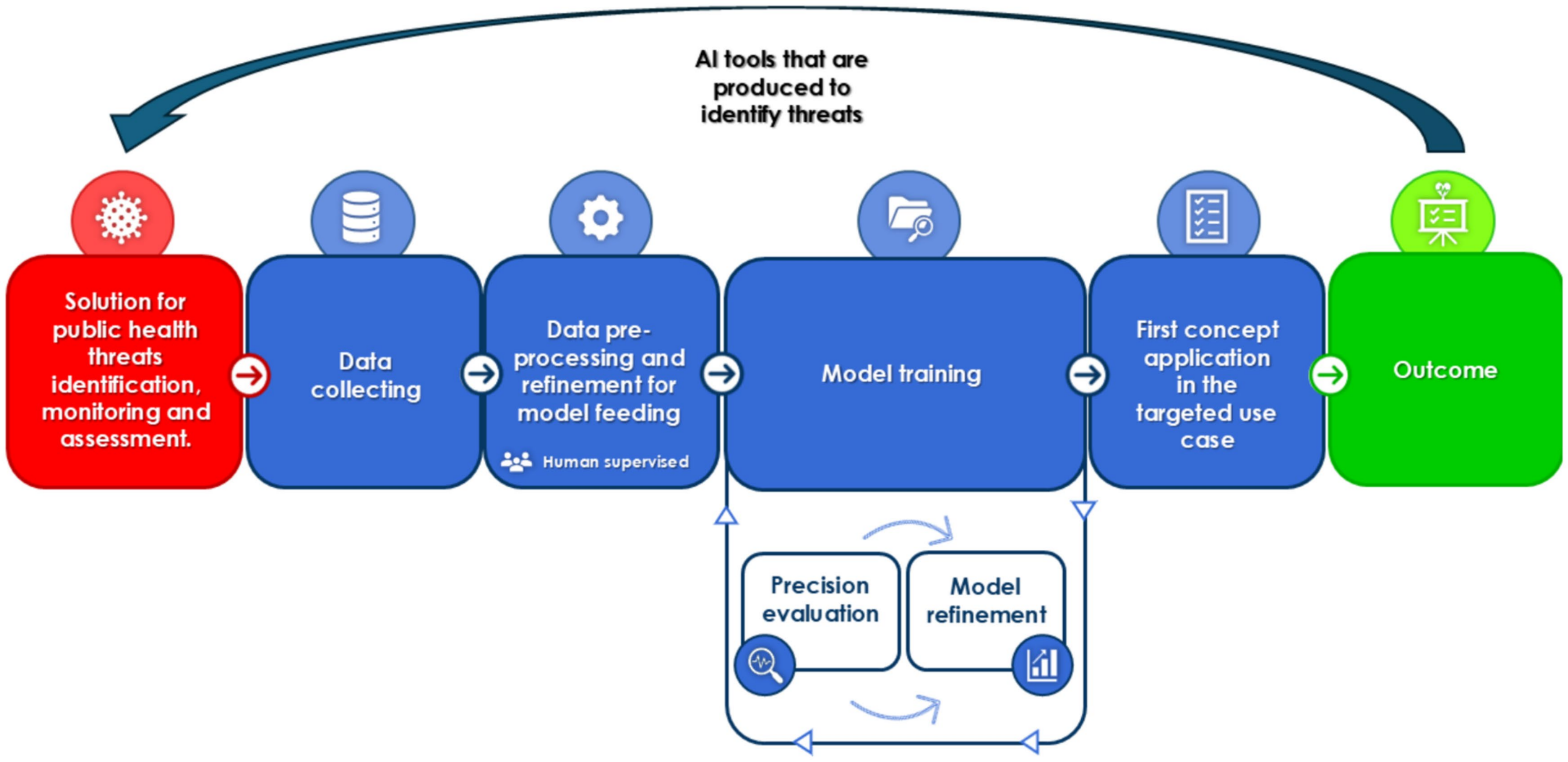
The processes involved in the development of a reliable AI based solution for public health threats identification and monitorization. Human intervention can occur at any step in order to maximize the effectiveness of the solution, but it is of paramount importance in data pre-processing and refinement to ensure the model is trained on verifiable and reliable data.

Artificial Intelligence (AI) is an increasingly transformative force in numerous different aspects of our daily life, and it has been of particular interest in the field of public health surveillance. It offers clear, unparalleled opportunities to leverage digital health solutions, with a clear translation toward health benefits and the promotion of better living for societies worldwide (10). AI-powered technologies allow the collection, analysis, and interpretation of vast and complex datasets, beyond the reach and ability of common public standard-of-use public health practices, such as contact tracing and diagrams, facilitating the possibility of timely and accurate detection of disease outbreaks, monitoring of health trends, and prediction of emerging public health threats and risks (11). By improving traditional surveillance systems, AI brings key benefits, including the optimization of decision-making processes and resource allocation, which are critical in managing applicable countermeasures (12, 13) and allowing AI to shape the public health field. Figure 1 shows the standard workflow when developing an AI solution for public health threats identification and monitorization, in order to achieve a full market release.

**Figure 2:**
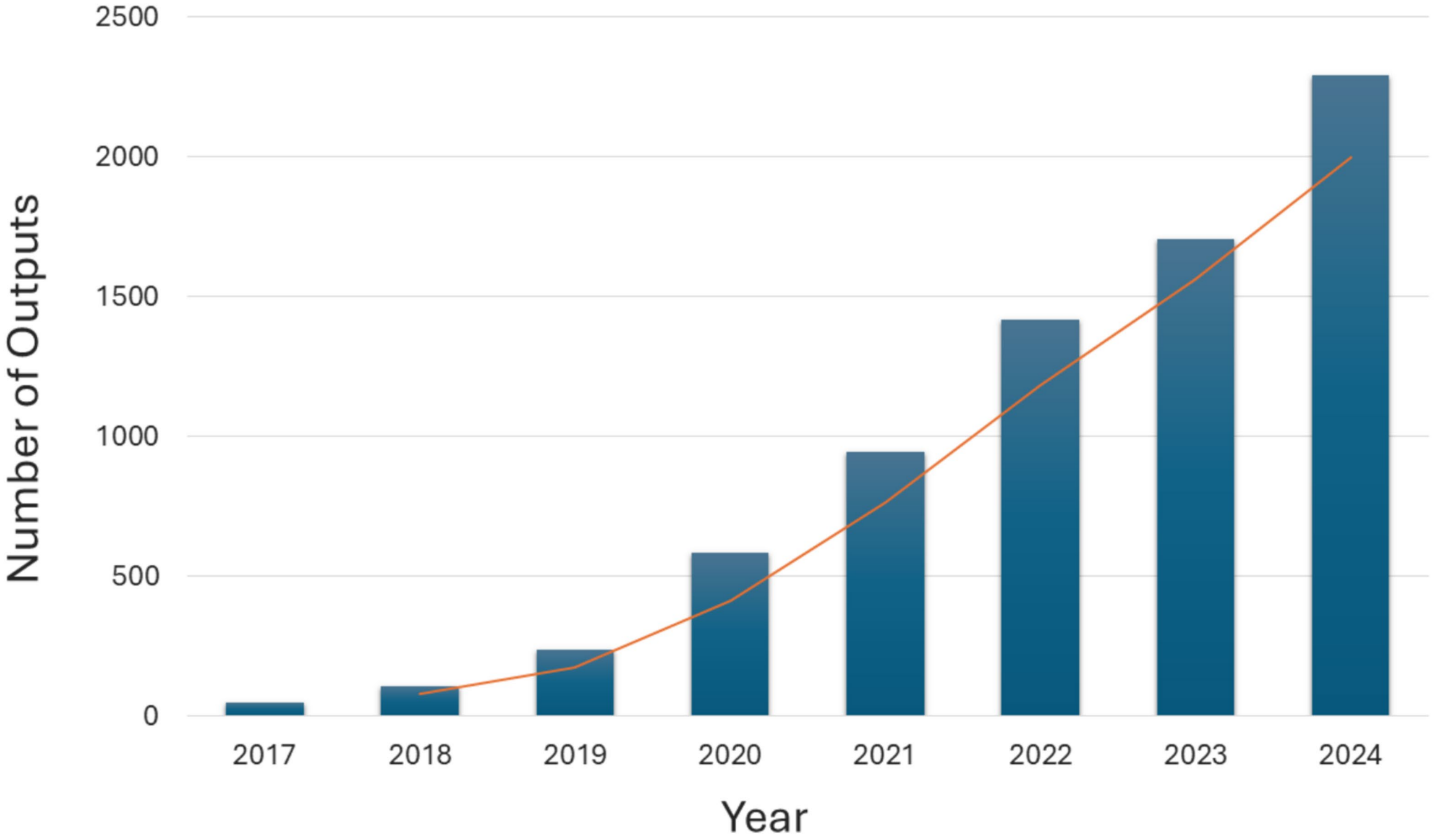
Number of outputs for the query “Public Health” and “Artificial Intelligence” in PubMed (2017–2024).

Current research in AI and public health surveillance is advancing rapidly (Figure 2). Most research has been focused on developing predictive algorithms, machine learning models, and natural language processing tools capable of real-time data analysis (13). As it currently stands, most efforts are being directed toward integrating heterogeneous data sources such as electronic health records, social media, environmental sensors, and genomic data to create a holistic view of public health dynamics. Concomitantly, research in the field of AI has great care in addressing challenges such as data privacy, bias in AI models, and the need for robust validation frameworks to ensure the reliability and equity of AI applications (14).

To continue innovating in this area, and to build upon this foundation, future developments in this domain aim to refine the interoperability and scalability of AI solutions, and to ensure their seamless integration into existing public health systems globally. Emphasis should be placed on fostering collaboration between multidisciplinary stakeholders, including public health authorities, data scientists, policymakers, innovators, and AI developers to align innovations with public health priorities. Ethical considerations, such as equity in access to AI-driven interventions and safeguarding data security, will also remain central to this agenda (15).

Based on the selected studies, the analysis presented below was organized into the following areas: (i) disease surveillance and prevention, (ii) disease modeling and outbreak management, and (iii) harnessing the internet and social media for disease surveillance in the digital world. Then, relevant AI standards and legislation were also discussed. This analysis aimed to explore the potential of AI tools for public health surveillance across these key areas.

#### 3.1.1 Disease surveillance and prevention

Disease prevention remains crucial for identifying, monitoring, and responding to emerging public health threats. While traditional techniques like clustering, phylogenetic trees, and outbreak trend analysis have been long utilized in public health surveillance, their scope is generally limited to smaller datasets, targeted applications and constrained for demographic models with a large geographical distribution. To counter these limitations, AI-driven tools, significantly enhance scalability and enable the analysis of vast, complex datasets, with moderate to high degrees of confidence while accelerating response time, making early disease detection more effective and with better odds of prediction (16). Among the available AI-driven tools (machine learning, deep learning, natural language processing, and large language models) machine learning models have been instrumental in early disease detection by analyzing vast datasets within a reduced amount of time, which would be infeasible using traditional methods alone (17, 18).

The integration of AI in public health can be leveraged to anticipate potential outbreaks, enabling early detection and response strategies that significantly minimize their impact on communities and healthcare services. This anticipation is vital for implementing effective public health measures to contain potential health threats before they escalate into epidemics or pandemics. Understanding and predicting the geospatial risk of outbreaks and the evolution and spread of health threats can further inform public health responses (19). In this context, AI methods can identify and predict the risk of outbreaks both geospatially and temporally, while preserving key information that carries important signals, and further contribute to the ability to predict locations that requires more care or aggressive measures to preserve public health and mitigate catastrophic events.

The main issues that arise when trying to develop adequate time series of epidemiological data often exhibit seasonality, non-stationarity, and sparsity, which pose significant challenges for accurate prediction. The ability to forecast such data has important public health implications, driving the development of both univariate and multivariate predictive models. These models rely solely on dependent variables for making predictions. As an example, the study by Wu et al. proposed a deep learning approach for the epidemic prediction of influenza from a time-series forecasting perspective using as basis datasets from Japan and United States (US) (20). In this study, natural language processing (NLP) techniques were applied using (i) convolutional neural networks (CNN), commonly used to capture local patterns in spatial data, to correlate information across different sources; and (ii) recurrent neural networks (RNN) to model temporal dependencies within data, as they are designed to process sequences by retaining memories of past inputs through recurrent layers/connections. To mitigate the risk of overfitting, a residual module was incorporated into the model architecture. When compared to traditional linear and non-linear methods, such as autoregressive and gaussian process regression methods, respectively, the proposed approach consistently outperformed these methods, demonstrating significant improvements in forecasting accuracy (20).

Spatial correlation can be applied to different geographic scales to enrich results. By using graph-structured recurrent neural network (GSRNN), Li et al. developed a model that took advantage of the spatial information, allowing data from the adjacent regions to introduce regional spatial features (21). For this study, the model used data sourced from the Center for Disease Control (CDC), which compiles information from around 100 public and 300 private laboratories across the US. As a result, the model achieved the same level of prediction accuracy of influenza spread and infection, while reducing the network weights by 70%, in comparison with standard benchmarks used by local healthcare companies, such as ARGO. Thus, this model showcases an opportunity to maintain epidemiological prediction abilities with AI, while also reducing both costs and processing time, allowing for faster forecasts (21).

Several of these scenarios take into consideration regular disease propagation and the infection rate that is calculated through the epidemiological mathematical formula. In reality, an outbreak of infectious diseases is highly correlated with external and non-dependent variables, which make prediction models much more complex, and create many more confounding variables that can significantly dampen the ability of AI models, greatly raising the confusion matrix (22). Commonly used external variables include climate data, such as temperature, humidity and air quality; search indexes; social media text data; and population migration, among other examples. The selection and weighting of these variables significantly influence prediction performance and are never consistent, depending on the demographics of the analysis (23).

For instance, the study by Chae et al. analyzed four types of data to predict malaria, chickenpox and scarlet fever in Korea—diseases subject mandatory reporting—including search query data, social media data, humidity and temperature (24). Optimal parameters were determined using a variable selection method based on ordinary least squares (OLS). Since the relationship between actual instances of disease occurrence and the internet search query data tends to have a time lag, a lag was added into each infectious disease-specific dataset to find future trends. The data was then analyzed using three different models: (i) deep neural network (DNN), (ii) long-short term memory (LSTM) and (iii) autoregressive integrated moving average (ARIMA). Finally, to compare model performance, the root mean square error (RMSE) was calculated. As relevant results, both the DNN and LSTM model showed better predicting capabilities (20–26% for chickenpox and scarlet fever), where the DNN model had a better performance all around, but LSTM showed more accurate predictions during periods when infectious diseases were spreading. As malaria had too few reported cases, the information was not as accurate, showcasing the need for accurate and strong data for the AI models. It should be noted however that the study had a short data collection period (1 January 2016 to 29 July 2017) and still employed a relatively narrow range of parameters for a deep learning model.

Hepatitis E has become a major public health problem. To address this problem, Guo et al. proposed the use of machine learning (ML) models, such as LSTM, support vector machine (SVM) and ARIMA, to predict monthly incidence and cases number of hepatitis E in China, using data from the Shandong Center for Disease Control and Prevention collected between 2005 and 2017 (25). To assess the effectiveness of the methods, the performance of each model was evaluated using three key metrics: RMSE, mean absolute percentage error (MAPE), and mean squared error (MSE). This study concluded that LSTM showed the most promising suitability for forecasting the monthly number of cases of hepatitis E, compared to ARIMA that struggled with a large prediction deviation. LSTM also had a better MAPE (13.6%) compared to ARIMA.

Metapopulation models are widely used mechanistic models that effectively capture spatial heterogeneity in disease dynamics and have been successfully applied to modeling and forecasting infectious diseases. The spread of infections in human populations is influenced by social contacts and individual mobility and, thus, researchers can utilize human mobility data to predict disease dynamics, and in cases where high-quality mobility data is unavailable, standard models like gravity or radiation can be used (26). Venkatramanan et al. developed a study focused on retrospectively forecast influenza activity within and surrounding the counties of NYC and Australia. In this study, their approach involved using a machine-learned anonymized mobility map to retrieve data from several key data sources, such as Google Aggregated Mobility Research Dataset, CDC FluView, EpiQuery NYC syndromic surveillance, positive flu results reported in New Jersey, Australia, and other data sources regarding demographics to allow correlation. Through the implementation of this model, the authors were able to identify likely locations for the next focal outbreaks. This study concluded that the model could be effectively used in predicting infectious diseases, particularly demonstrating improved performance during the early weeks of an outbreak and primarily serving as a method validation process. This study also highlighted that high-quality mobility data can significantly improve disease modeling and forecasting, particularly in regions with detailed surveillance systems. However, mobility data present limitations, such as difficulties in distinguishing trip durations and differentiating between residents and transients. Further refinement of aggregation methods and the integration of multiple mobility models could enhance forecasting accuracy (26).

Understanding the distribution of Aedes mosquitoes can help prevent and predict human arbovirus infections, such as Zika, Dengue and Chikungunya. In 2018, a study was conducted by Ding et al. using multidisciplinary datasets—including occurrence records, social factors, and meteorological factors—to train ML models for simulating the global distribution of *Aedes aegypti* and *Aedes albopictus* (27). The models applied included SVM, gradient boosting machine (GBM), and random forest (RF). The results showed that RF achieved the highest accuracy value (with an area under the curve of 0.973 and 0.974 for the two mosquito species analyzed, respectively), indicating highly precise predictions of both mosquito species’ distributions. However, it should be noted that RF showed statistically significant differences only when compared to SVM, while GBM showed no significant differences based on the validation datasets. Among the factors analyzed, temperature suitability was found to have the strongest discriminatory dominance.

RF was also used to assess the risk of dengue transmission in Singapore in 2018 (28). The study by Ong et al. analyzed past dengue exposure data from 2006 to 2013. The model incorporated epidemiological, demographic and environmental factors, such as (i) dengue exposure indicators (number of cases in previous year, the neighboring total cases in previous year and number of non-resident cases in previous year), (ii) estimated human population density, (iii) vector population (estimated ratio of *Aedes aegypti* mosquitoes out of all Aedes mosquitoes, counting on the breeding percentage) and (iv) environmental data (vegetation index, connectivity index and ratio of residential area). The RF model ranked the overall risk of dengue transmission across different areas for a given year and color-coded the ranks into risk groups on a map. Using the latest available information regarding dengue cases, the model demonstrated that their prediction matrix could confidently predict around 80% of risk areas (using an 80% prediction interval). There was also a high correlation (>86%) predicting high risk areas and likely dengue cluster outbreaks. These findings were important for prioritizing vector control efforts, significantly supporting the Singapore National Environmental Agency in allocating limited resources more effectively, focusing on areas with the highest transmission risk.

With the goal of improving existing models, namely the LSTM, Liao et al. conducted a study focused on the development of a time-dependent model to predict COVID-19, called Susceptible, Infected, Recovered, Vaccinated, and Deceased-Deep Learning (SIRVD-DL). The model combined mathematical models of infectious diseases and was incorporated into a deep learning model, to overcome the limitations associated with deep learning models regarding development trends. The authors used two main data sources, (i) the Johns Hopkins University System Science and Engineering Center, which includes global cumulative confirmed cases, cumulative cured cases, and cumulative deaths, and (ii) Our World website, that includes data such as confirmed cases, deaths, hospitalizations, testing, and vaccinations. The study showed that this model could both sufficiently predict infection rate and address recovery and death rate from confirmed cases, giving it the potential to be used both as a modeling tool and as a prediction tool. It had a 51% improvement in single day prediction compared to other deep learning models, while also displaying short and medium-term predictions with error rates of only 5.07 and 10.93% (29).

Integrating genetic data with AI in public health surveillance has the potential to improve the detection and control of communicable diseases by enabling precise tracking of pathogen transmission and evolution, as well as supporting the identification of therapeutic options in potential outbreaks. AI’s advanced analytical capabilities can facilitate the rapid processing of complex genetic information, improving response strategies and intervention efforts. For instance, the study by Al-Amran et al., showed how AI can also be used to analyze both common or emerging pathogens by analyzing their genomic profile and assessing the likelihood of genetic mutations and of certain pathogens toward potentially pandemic viruses. By integrating data from zoonotic reservoirs, human-animal interfaces, and environmental factors, and using publicly available genome databases (GenBank and GISAID), the authors employed deep learning models, including CNN and RNN. Their findings demonstrated that, after rigorous training with these datasets, these models could forecast viral mutations, assess potential virulence and adaptability, and estimate the likelihood of spillover event, helping to identify zoonotic risks and potential pandemics. While further analysis is needed, this preliminary study sheds light of AI potential as a useful tool for early detection of virus transformations (30).

In another study conducted by Gill et al. (31), two pre-trained large language models (LLMs), called BioBERT and BERN2 that made up a framework termed Gene Interaction Extraction (GIX), were used for automated extraction of genetic interactions from unstructured data. These models were specifically designed to process biological data and extract complex relationships between entities such as genes and proteins from scientific literature, which would simplify the process of identifying therapeutic options in potential outbreaks. The proposed framework showed potential for improving public health surveillance by automating the extraction of relevant information from massive biomedical datasets, a task that is traditionally slow and resource intensive, and managed to process over 1,000 abstracts within a single scan of the LLMs. This type of analysis and process interaction is of paramount importance as it allows research efforts in outbreak response to avoid research bottlenecks.

These recent examples of academic work highlight the promise of ensemble forecasting, which combines the outputs of different AI models to potentially improve predictions in the public health contexts. However, to optimize this approach, good practices must be established to ensure stable and reliable operational performance in future forecasts (32). As it currently stands, AI tools can be used to moderately forecast potential focal hotspots for diseases, which would allow public health measures to be undertaken in a preventive manner rather than in response to a health risk or emergency.

#### 3.1.2 Disease modeling and outbreak management

To implement an AI solution for predicting an outbreak evolution, several factors must be considered, such as processing large multi-dimensional data to detect early warning signals, identifying local and regional patterns, cross-referencing demographical and geographical trends, modeling and simulating outbreak behaviors and detecting misinformation and disinformation that could hinder pandemic responses (33). The main challenges lie in achieving sufficiently high accuracy in a timely manner while processing these multiple variables listed above without compromising accuracy (19).

In fact, while COVID-19 first cases presenting severe pneumonia were officially reported in China approximately on December 8, 2019, a retrospective study by Kpozehouen et al., using open-source intelligence data available at EpiWatch’s ML analytic systems, and data regarding hospital admissions and treatment courses identified in Google and Chinese Banu, identified COVID-19 cases in China in mid-November 2019, suggesting that the outbreak had begun earlier than what most modeling estimations were assuming (34).

Although early detection of outbreaks is key, there are cases where preventive measures are only applied when the disease is already established and has reached community-wide spreading. Herein, AI technologies can help model disease propagation and epidemic spread, enabling public health authorities to design and implement effective interventions measures. To accomplish this, AI models must be capable of capturing complex dynamic systems with non-linear relationships. Additionally, they should effectively handle time-series data, accounting for lags between interventions and responses, while ensuring accurate predictions of the disease behavior at a community-wide level (35).

Complex network and agent-based computing models are two widely used methods in this context. Complex network models can vary significantly in computational complexity depending on their design and implementation, with some offering high-level abstractions and lower computational demands, while others, especially those involving detailed interactions or large-scale simulations, may exhibit substantial computational complexity (36). Agent-based methods have higher data resolution and can flexibly reconstruct detailed plans and reproduce the complex process of disease transmission, which is necessary to assess major responses and interventions in real-world settings, but can struggle as the number of agents increases, as the time for simulation might grow exponentially. This section examines various studies that have used AI models for disease modeling, highlighting their potential to enhance public health threat management.

The LSTM model was applied for predicting the trend of COVID-19 pandemic in Canada (37). In this study conducted by Chimmula et al., the LSTM model achieved an accuracy of approximately 93%, enabling the prediction of a potential peak within 2 weeks, and estimating the potential outbreak’s ending point around June 2020. This study also revealed that the public health policies implemented by Canadian authorities to minimize the human exposure had a positive impact, especially when compared to the responses of other countries such as the US and Italy. In fact, following the success and the capabilities of the model, in Bangladesh, LSTM was the chosen ML model to predict the COVID-19 evolution (38). While the LSTM model showed a high accuracy in predicting cases numbers, its accuracy was medium to low particularly for long-term predictions of death cases, suggesting room for further improvements in the model’s applicability.

Moreover, in 2022, a study by Li et al., developed a novel method based on auto-reservoir neural network (ARNN), a type of recurrent neural network with a fixed multilayer architecture, to detect early warning signals of COVID-19 transmission in six countries and regions. The study analyzed short-term, high-dimensional time-series data from 2020 to 2021, and processed it through the ARNN-based Landscape Network Entropy (ARNN-LNE) index (39). This approach successfully identified early warning signals of a disease outbreak before a critical transition from a normal state to an outbreak state. This method was validated in all the regions tested, with a primary focus on Germany, Italy, Netherlands, Spain and Canada, while also considering additional countries of the Eurozone. For example, this model identified a yellow warning signal on July 3, 2021, while the German disease control agency defined the outbreak stage on August 20, 2021. Moreover, the model was compared with the Support Vector Regression (SVR) for predicting daily new COVID-19 cases, showing that ARNN outperformed SRV in forecasting, achieving overall lower RMSE in the six datasets. On the other hand, the ARNN-LNE method also outperformed the SVM model in early COVID-19 warning classification, achieving an area under the curve (AUC) of 0.825 compared to 0.77 for SVM.

Still concerning COVID-19, a study by Wang et al., was undertaken to predict daily new cases and cumulative confirmed cases in the US, Brazil and India, over a 30-day period. The study used as basis a dataset published by the WHO of new and cumulative confirmed cases of COVID-19 (40) and evaluated three ML models for this purpose: ARIMA, Seasonal ARIMA (SARIMA) and Prophet, an open-source automated ML. The performance of the different models was evaluated using RMSE, mean absolute error (MAE) and MAPE. The results indicated that the Prophet model performed best in predicting COVID-19 case trends in the US, due to its architecture based on ML fitting, which could automatically allow it to estimate long-term predictions, whereas the SARIMA model showed a tendency toward overfitting in this context. For the prediction of cumulative cases, the ARIMA model showed a stronger ability to fit and forecast data with a growth trend, particularly in Brazil and India. These findings indicate that such predictive models can support proactive measures and policy development for better epidemic management.

Another significant example to model COVID-19 cases is the Differential Equations Lead to Predictions of Hospitalizations and Infections (DELPHI) model, as described by Li et al., that extends the classical Susceptible-Exposed-Infectious-Recovered (SEIR) framework by incorporating additional compartments such as undetected, hospitalized, and quarantined cases. This model accounts for the dynamic effects of government interventions through a time-dependent contact rate, enhancing its predictive accuracy. However, it is a closed system as it does not include demography (births, non-COVID-19 deaths) or migration. This model has been incorporated by the CDC, in their core ensemble forecast and has been utilized by different health and federal agencies for the pandemic planning. This model employs differential equations to predict, with moderate accuracy, the effects of underreporting cases and the impact of governmental intervention measures on the outbreak progression, while also identifying potential future outbreak hotspots. It was able to predict endemic events with a high degree of confidence 1–2 months before and was used by vaccine developers to pinpoint optimal locations to run the clinical trials, to streamline the development of the vaccines. However, being mostly a deterministic model, it is not well-suited for propagation and quantification tasks, which are essential for theoretical scenarios and predictive analysis. It also does not account for physiological differences across population cohorts (sex, age, occupation), and models most patients based on standard time-to-recover from the infection, not considering cases with prolonged infections (41).

To detect new dengue cases and possible outbreaks, a study done by Benedum et al. compared the performance of different ML algorithms (RF, Random Forest-Univariate Flagging Algorithm (RF-UFA), and ARIMA) with that of traditional linear regression models [Generalized Linear Models (GLM) and Generalized Additive Models (GAM)] (42). For this study, weekly dengue cases and meteorological data from three cities (Iquitos of Peru, San Juan of Puerto Rico, and Singapore) was used spanning various time frames, such as 1990 to 2010. ARIMA seemed to perform optimally when considering long-term dengue outbreak forecast since it captured broad transmission dynamics and seasonal trends, but only in Singapore, since it struggled to differentiate between small- and large-scale transmission events. When we consider short-term forecasts of weekly case counts using surveillance data, RF-UFA ML models demonstrated 21 and 33% lower error rates compared to regression and time series models, respectively. However, when relying solely on weather data, these ML models did not show a meaningful improvement. In predicting weekly dengue outbreaks up to 12 weeks ahead, ML models attained a maximum Matthews correlation coefficient (MCC) of 0.61.

ML models were applied to predict the R_0_ for disease spread, a key metric that indicates the controllability of the disease spread (43). The study used both simulated and real-word network data and employ two primary ML regression models, SVR, known for its effectiveness in managing non-linear relationships, and Artificial Neural Networks (ANN), designed to capture complex patterns within data. These models were trained using six structural properties of the networks: average degree, average shortest path length, clustering coefficient, network density, network diameter, and maximum degree. The study showed that both SVR and ANN models can predict R₀ with high accuracy across various network types. The training process is computationally efficient, representing a one-time investment in terms of time and memory. Once trained, these models can predict R₀ for new, unseen networks, demonstrating their generalizability. This approach underscores the significant role that network structure plays in disease spread and highlights the efficacy of ML regression techniques in epidemiological modeling. This implies that it is possible to predict the value of R_0_ at the very first stage of obtaining the test network, without waiting until the epidemic outbreak is completed or reaches a stable state.

In 2022, Cardoso et al. published an article exploring a method for modeling the spread of COVID-19 using spatio-temporal convolutional sequence-to-sequence neural networks, based on the STConvS2S architecture (44). The authors assessed the effectiveness of this approach using real-world data obtained during the first year of this pandemic in Portugal. The proposed method performed better than the other tested alternatives (VAR, ARMA, SIRD), resulting in lowest standard deviations in predictions ranging from 7 to 14 days ahead, using RMSE and symmetric mean average error (approximately 2-3-fold reductions). The methods were based upon a simple cell level autoregressive moving average (ARMA) model, a cell level vector autoregressive (VAR) model, and a municipality level compartmental SIRD model, which followed by the same geostatistical simulation method used to generate the reference data. The experiments showed that the convolutional sequence-to-sequence neural network provided the best results in terms of predictive accuracy. Furthermore, the predictions performed by this method showed a gradual deterioration in performance as the prediction horizon extended into the future, highlighting the importance of spatio-temporal information in pandemic predictions.

Additionally, Ardabili et al. performed a comparative analysis of ML models and soft computing techniques, to model and predict the COVID-19 outbreak (45). The modeling of this outbreak has differences with other recent infection diseases, especially due to the strict measures enforced by authorities, in which susceptibility to infection has been manipulated dramatically. As individuals committed voluntarily quarantine and limited their social interaction, standard models such as susceptible-infected-recovered (SIR) and susceptible-exposed-infectious-removed (SEIR) cannot present promising results, as gaging susceptible individuals is not feasible due to asymptomatic infections and sub-clinical infections that were not reported. In this study, specific DL techniques were used, such as Multi-Layered Perceptron (MLP) and Adaptive Neuro Fuzzy Inference System (ANFIS), for the modeling of the outbreak in five countries (Italy, Germany, Iran, US, and China). Results indicate that both daily and weekly sampling can be used in ML modeling and both models showed the progression of the outbreaks allowing the extrapolation for long-term prediction of COVID-19 disease up to 150 days. This study highlights the potential of ML models as effective tools to model the time series of the outbreak without the assumptions that epidemiological models generally require.

Furthermore, the study by Gomez et al. developed an agent-based model labeled INFEKTA and employed it to the COVID-19 pandemic, with a particular focus on Colombia (46). This model consisted of five key components, namely space, time, individuals, infectious disease dynamic and social distancing policy. The model represented Bogotá as a network of homes, workplaces, schools, markets, and public transport stations, where agents interact daily based on predefined probabilities for infection and recovery. The simulation evaluated various social distancing policies, ranging from no restrictions to medium (40% closures) and extreme (80% closures). The findings indicate that medium restrictions significantly reduced transmission without fully halting activity, while extreme measures suppressed cases but risked secondary waves due to residual susceptibility. The model’s alignment with real-world case distributions validated its utility, suggesting that INFEKTA could be used as a valuable tool for tailoring public health interventions. Furthermore, it highlighted the potential of agent-based modeling in supporting informed decision-making in infectious disease management.

The study by Nitzsche et al. utilized an agent-based model (ABM) to evaluate the effects of different lockdown scenarios and event characteristics on the transmission of SARS-CoV-2. The model simulates an abstract city of 100,000 agents, incorporating detailed SEIR disease progression states, agent interactions, and mitigation measures such as masking and social distancing. This study demonstrated that the model was able to predict the effect of several measures on the infectious rate, namely stricter lockdowns significantly reduce infection rates, with “Scenario III” (closure of schools, workplaces, public spaces, and primary care) achieving virus extinction in 40% of simulations over a 49-day lockdown. Conversely, less restrictive measures, such as “Scenario I” (masking and distancing without closures) and “Scenario II” (only primary care is open) resulted in secondary waves and higher cumulative infections. Events, modeled with variable size, frequency, and duration, contributed disproportionately to infections, for instance, events without social distancing accounted for approximately 29.4% of infections, compared to 16.8% when distancing measures were applied. Additionally, individual infection risk increased by 21% at events where individuals wore masks and maintained social distancing, compared to 48% at events where neither masks nor social distancing measures were observed, emphasizing the role of preventive measures. The study also highlighted the linear relationship between event duration and individual infection risk, while event size showed no significant effect. These findings underscore the importance of early, comprehensive lockdowns and targeted restrictions on high-risk activities, such as large events, to manage pandemic spread effectively, but also highlighted how close to reality these models can simulate the impact and the development of a disease that has hit community-wide spread status. This particular model seems to be tailored to being rapidly adaptable, which makes it ideal for fast and early decisions on lockdown scenarios (47).

The study conducted by Proctor et al. utilized frameworks from OpenAI’s ChatGPT-4 to create an AI-powered assistant designed to make infectious disease modeling more accessible. By interpreting natural language inputs, this tool allows users to generate or modify disease model configurations intuitively. It integrated with the open-source Compartmental Modeling Software (CMS) framework, facilitating a seamless experience from model setup to simulation and analysis. Moreover, the AI assistant could create model files, run simulations, interpret disease model parameters, and help interpret results. This study suggested that such AI assistants could significantly contribute to global health efforts by empowering researchers, especially in regions with limited resources, to develop and refine disease models independently. This approach has the potential to democratize disease modeling, offering a scalable solution adaptable to diverse needs across various geographies, languages, and populations. However, this study remains a prototype that would still require validation working with large complex data sets (48).

These studies demonstrate the potential of AI models to support disease modeling and outbreak management by enhancing the accuracy, efficiency, and timeliness of epidemic response strategies. These models can enhance the understanding of disease dynamics and support policymakers and health professionals in optimizing resource allocation and implementing targeted public health interventions. However, from these examples, it is crucial to identify the underlying causes of infectious disease transmission and spread to control disease evolution and prevent pandemics. Identifying the factors driving the spread of diseases can empower policymakers to make more effective informed decisions, such as forecasting vaccine needs and managing the respective purchase, designing targeted public awareness campaigns, and planning training programs for health professionals.

#### 3.1.3 Harnessing the internet and social media for disease surveillance in the digital world

Internet-based surveillance systems are becoming valuable in the surveillance of public health events that otherwise rely exclusively on laboratory diagnostic capability and timely notification by health professionals. It is characterized by providing unstructured information from multiple origins, such as search queries, social media, news, discussion forums, websites, and web encyclopedias. If mined regularly, these data could provide early signals of epidemics before official detection by health authorities using traditional approaches (49).

There are several systems that provide open access to epidemic intelligence, but not all of them currently employ AI technologies. Despite this, there has been a growing trend toward the introduction of AI tools in these repositories, to streamline the process of data processing and acquisition.

One such example would be Epidemic Intelligence from Open Sources (EIOS), which is a cutting-edge surveillance system developed by the WHO to leverage the vast array of information available on the internet and social media for global public health monitoring (50). EIOS is specifically designed to enhance traditional disease surveillance systems by incorporating AI technologies, such as NLP and ML (namely RF to n-grams or Google’s Bidirectional Encoder Representations from Transformers (BERT)), to automate the collection, classification, and analysis of publicly available data. This innovation enables the rapid identification of potential health threats, thereby supporting timely and effective responses (19).

The EIOS system processes a continuous flow of information from various open sources, including online news outlets, blogs, forums, and social media platforms, scanning thousands of articles and posts each day. By employing AI, the system can efficiently filter irrelevant data and prioritize reports with potential public health relevance. NLP algorithms are central to EIOS, allowing the system to understand context and extract key insights from large volumes of unstructured textual data in multiple languages. ML models further refine this process by identifying patterns and trends indicative of emerging health threats and can be used in large scale world events to give decision makers the ability to understand and predict certain risks (51, 52). AI’s role in EIOS extends to real-time monitoring, where it categorizes health-related events and geolocates potential outbreaks, as was employed by the WHO during COVID-19 (53). Additionally, the use of AI allows EIOS to adapt to evolving patterns of information dissemination, such as the increasing reliance on social media for reporting health-related incidents and aims to try to centralize reliable information on a single platform in order to limit the damage misinformation can cause (54). EIOS’s AI-powered system has been instrumental in improving the speed and accuracy of epidemic intelligence, aiding global efforts to prevent and control outbreaks. By automating the early detection of anomalies in health-related data, EIOS enhances situational awareness for public health professionals, enabling them to make informed decisions in response to emerging threats.

BlueDot is a web-based commercial program that harnesses AI to process and analyze big data from a wide range of official and unofficial sources in over 60 languages (55, 56). The platform integrates several datasets, including official health notifications, global media, and scientific, mobility, travel, and governmental data, among other examples. Additionally, BlueDot gathers data on global infectious disease alerts, real-time climate conditions, and vectors such as insects and animals that act as disease reservoirs. By combining these extensive datasets, BlueDot employs advanced AI-driven filtering and clustering tools to identify areas of interest, such as hotspots, cold spots, and spatial outliers. The AI models within BlueDot allows for rapid processing and synthesis of data, enabling the platform to detect anomalous disease events, assess associated risks, and predict potential destinations most likely to experience outbreaks (57, 58). Regular alerts are sent to clients, providing actionable insights to mitigate risks and informing public health responses. A notable example of BlueDot’s predictive power occurred in 2020, when its AI algorithms detected the emergence of COVID-19 in Wuhan, China, 9 days before the official announcement of the outbreak (59, 60). By analyzing real-time air travel data alongside other datasets, BlueDot not only identified the outbreak but also accurately predicted the city’s most at risk of experiencing the virus spread. BlueDot, through AI, has transformed the public health surveillance landscape, enabling quicker detection and response to infectious disease threats. By integrating diverse data sources and advanced computational tools, AI can significantly enhance global health, by enabling faster responses, improved preparedness, and more effective interventions.

EpiWatch, developed at the University of New South Wales, Sydney, is a web-based epidemic surveillance application enhanced by ML (prioritization algorithms, NLPs) and other AI technologies (such as custom-made LLMs developed based upon openAI’s API and Chat-GPT4). Launched in 2016, EpiWatch leverages AI to collect and process outbreak data from a wide range of sources, including media reports, press releases, official government reports, and social media. Using NLP and ML, the system automates data collection and analysis, allowing it to identify signals of emerging epidemics quickly and efficiently than traditional surveillance methods. As an open-source epidemic observatory, EpiWatch provides a global map and a searchable, sortable, and filterable table of epidemics, offering insights with data visualization options for up to 30 days. AI technologies enhance the system’s ability to process large volumes of information in multiple languages, identifying outbreak patterns and risk factors that might otherwise go unnoticed. These capabilities significantly reduce the time required to detect and respond to potential epidemics, enabling public health officials to take early preventive measures (61). EpiWatch is further strengthened by specialized tools that expand its utility. These include Flucast, a seasonal influenza forecasting tool; Epirisk, an epidemic risk analysis tool; and ORIGINS, a tool designed to determine the origins of epidemics. Together, these tools provide comprehensive insights into outbreak trends and risks, supporting better decision-making and resource allocation in public health (62, 63).

In 2020, Puca et al., used EpiWatch to monitor global mumps outbreaks from 2016 to 2019. During this study, EpiWatch was able to identify cases that were not reported to the WHO and pinpoint focal hotspots (e.g., universities), while also allowing full contact tracing of the spread of the hotspot, allowing the authors to trace its origin. This study demonstrates the usefulness of EpiWatch to control the spread of health threats (64).

During the COVID-19 pandemic, methods leveraging social media, which were validated in previous outbreaks, were applied prospectively to inform advisories of both healthcare practitioners and public health administrators. Social media reactions, especially on Instagram and Twitter, can serve as proxies for outbreak monitoring and evaluating the effectiveness of public health interventions. Although social media has the potential for positive public health utility, it can also amplify poor quality content and perpetuate the spread of misinformation. Public fear and anxiety are known to be heightened by sensational reporting in the media during outbreaks, a phenomenon highlighted by the ease of sharing on social media (65).

Serban et al. reported the development of a software system titled SENTINEL for real-time syndromic surveillance (66). This system integrates multiple data sources, including Twitter (now X) and CDC reports, to detect symptom report spikes, and generate real-time situational awareness for public health monitoring. SENTINEL primarily relies on Twitter data for its Event Detection and Nowcasting systems, using ML (DNN models, CNN and LSTM) to classify tweets and news reporting illness symptoms and estimate real-time disease prevalence. The authors also implemented a SVM model using the LibShortText toolkit and a Multinomial Naïve Bayes model using Scikit-learn to serve as baselines, both using term Frequency-Inverse Document Frequency (TF-IDF) feature vectors. The study demonstrated that neural networks consistently outperformed baseline methods, with both CNN and RNN achieving higher F1 scores than SVM and Naïve Bayes across news and Twitter classification tasks. In Twitter classification, CNN achieved the highest F1 score of 0.852, significantly surpassing Naïve Bayes (0.735). For news classification, RNN outperformed all models with an F1 score of 0.939, compared to 0.814 for Naïve Bayes. Twitter provides timely, high-coverage, and publicly available data, but challenges such as noise, low confidence, and demographic bias require mitigation through health-related tweet classifiers and integration with news sources. For that, the Nowcasting algorithm combines past CDC reports with current aggregated Twitter symptom data to forecast disease levels, addressing the 1–2 week reporting lag in official surveillance. News data is used as a secondary source to validate or adjust confidence in Twitter-based outbreak detection, drawing from global, national, and regional health news sources.

Another study by Espinosa et al. explored the use of LLMs to extract public stances toward vaccination from social media posts. Vaccination status is a key marker of public health, and the growing distrust in the effectiveness of vaccines due to the widespread of misinformation in social media has been identified as a worldwide public health threat (67). The study analyzed 1,000 English-language vaccination-related tweets (2019–2022) using six classification methods, including experts, crowdsourced annotations, and four LLMs (GPT-3.5, GPT-4, Mistral 7B, and Mixtral 8x7B). In addition, the authors used Valence Aware Dictionary and sEntiment Reasoner (VADER), a rule-based sentiment analysis tool, to classify the general sentiment as a baseline. The findings suggest that LLMs performed better than crowd annotators, particularly in the negative stance classification, which had the lowest F1 scores and sensitivity across all methods. GPT-4, Mistral (7B) and Mixtral (8 × 7B) showed a less prompt-dependency than other models, providing consistent results across different settings. All methods performed worse on tweets with partial expert agreement, highlighting challenges in resolving ambiguity due to polysemy, lack of context, sarcasm, and misunderstandings in short social media texts. This emphasizes the difficulty of objective classification and the need to account for misclassification risks in scalable methods.

Similarly, the study by Argyris et al., aimed to examine the varying discourse among pro- and anti-vaccine populations, using a supervised classification algorithm to classify the tweets (K-means), followed by unsupervised clustering algorithm (logistic regression classification) and a multistep qualitative analysis to identify the discursive topics, and how vaccines are framed (pro-vaccine, anti-vaccine or neutral). The study found that anti-vaccine discussions generally had a higher inter-topic distinctiveness (as in, topics that were far too different from the vaccine topic) and tend to generate a more persuasive and fearmongering discourse to captivate a response. In contrast, pro-vaccine discussions lacked a clear problem statement, leading to poor population engagement (68).

Moreover, a study by Harris et al., evaluated the use of LLMs for public health tasks, focusing on classification across three areas (burden, risk factors, and interventions) and extraction from free text (academic, news, social media, and questionnaires) (69). The authors combined six externally annotated datasets with seven internally annotated datasets to assess five open-weight LLMs ranging from 7 to 70 billion parameters (Llama-2-3, Mistral, and Flan-T5 base models) using zero-shot in-context learning. The findings indicate that Llama-3-70B-Instruct was the top performer, achieving the highest micro-F1 scores in 15 out of 17 tasks. Performance varied across tasks, with all open-weight LLMs surpassing 80% micro-F1 on tasks such as Gastrointestinal Illness Classification but scoring below 60% in more complex tasks like Contact Classification. Additionally, in a subset of 12 tasks, GPT-4 was evaluated, showing comparable results to Llama-3-70B-Instruct, with each model outperforming the other in 6 of the 12 tasks. These findings suggest that LLMs hold promise as tools for public health experts to extract information from diverse free text sources, potentially enhancing public health surveillance, research, and interventions.

While social media data offers valuable insights for public health assessment, it has several drawbacks, such as (i) health-related terms may not always refer to actual illnesses; (ii) users may misreport conditions (e.g., flu vs. common cold), and (iii) there is demographic bias, as younger individuals are generally more active on social media (70). Monitoring social media and public opinion may be necessary to effectively prevent and control potential epidemics. Therefore, relevant authorities can strengthen the filtering of the network environment with the help of AI technology, to monitor and control false reports on public health events, guide public opinion, and improve the response and governance ability to manage public health emergencies. As AI technology advances, the integration of these models into public health surveillance systems could substantially improve the ability to monitor and respond to changing public health attitudes (71).

In summary, accurate and reliable predictions of infectious diseases are invaluable to public health organizations for planning interventions to reduce or prevent disease transmission and mitigate the negative impacts of outbreaks (72). Estimating diseases trajectories and patterns allows timely preventive measures, like schools’ closures, borders restrictions, public service suspensions, and effective medical countermeasures distribution. However, predicting the emergence and spread of outbreaks is challenging due to their sporadic nature and limited data on key transmission parameters, such as transmission pathways, immunity level and duration, which are crucial for building realistic epidemiological models (73).

The AI technologies discussed in this review clearly demonstrate their ability to generate early epidemic warnings without relying solely on passive human reporting, enabling timely intervention and management of emerging outbreaks. AI can also help to address challenges like limited healthcare infrastructures and human resources, particularly in low-income countries, where early detection is essential to prevent global spread. Comparing epidemic prediction studies is challenging, but most studies have reached similar conclusions, showing that AI models can be used to effectively monitor and predict major infectious disease outbreaks. Moreover, integrating different AI models has been shown to enhance the accuracy of traditional epidemiological models. Therefore, combining multiple AI models with various data sources can significantly improve spatial and temporal predictions of infectious disease trends and incidence. However, public health institutions may need guidance in selecting models that align with their specific goals, as well as frameworks for continuous accuracy assessment and monitoring to adjust strategies as new technologies and scientific knowledge emerge. Given the little-to-no margin of error, addressing confidence intervals, data reliability and predictive precision is crucial in driving AI innovation for public health applications. To maximize the impact of AI-driven disease modeling and outbreak management, integrating it into a One Health approach is essential, recognizing the interconnection between human, animal, and environmental health, and promoting cross-sectoral collaboration (74, 75).

#### 3.1.4 AI principles, standards and legislation

The rapid advancement and integration of AI across multiple sectors has introduced several advancements, such as data management, resource allocation and population health management (76, 77). However, this progress also presents critical ethical, legal, and technical challenges that require robust standards and regulations to ensure safety, equity, and privacy in its implementation. This is particularly crucial in public health, a field where AI holds transformative potential as shown by the several studies examined in this article. This section summarizes key standards and regulations, with special focus on EU-level frameworks, that are essential for fostering the responsible and effective use of AI in public health. However, it does not provide an exhaustive list of all applicable regulations, such as the General Data Protection Regulation (GDPR) and Medical Device Regulation, among others.

##### 3.1.4.1 OECD recommendations for responsible use of AI in health

In May 2019, the OECD established the *AI Principles*, which serve as a global benchmark to guide governments, organizations, and individuals toward responsible AI development (78). These principles, endorsed by over 50 countries, emphasize the importance of (i) promoting inclusive growth, sustainable development and well-being; (ii) respecting human rights and democratic values, including fairness and privacy; (iii) ensuring transparency and explainability; (iv) maintaining robustness, security and safety of AI throughout its lifecycle; and (v) holding organizations and individuals accountable for adherence to these principles. They also highlight the necessity of international collaboration to avoid fragmentation and inequities in AI implementations. By promoting trust and capacity building, these principles aim to enable AI-driven improvements in health systems while minimizing potential risks.

The OECD also published in 2025 the *Collective Action for Responsible AI in Health*, which underscores AI’s potential to revolutionize healthcare by enhancing equity, resilience, and sustainability (79). However, the report identifies significant barriers to the effective adoption of AI, such as inadequate data governance and prevailing low trust in AI systems. To address these challenges, the report proposes actionable steps to advance responsible AI adoption, including fostering trustworthiness, building capacity, enabling adaptability, and promoting international collaboration.

##### 3.1.4.2 WHO ethics and governance of artificial intelligence for health

In 2021, the WHO published the report *Ethics and Governance of Artificial Intelligence for Health*, which delineates six ethical principles essential for the design and implementation of AI systems, which includes (i) protecting human autonomy, (ii) promoting well-being, human safety and the public interest, (iii) ensuring transparency, explainability and intelligibility, (iv) fostering responsibility and accountability, (v) ensuring inclusiveness and equity, and (vi) promoting AI that is responsive and sustainable (80). These guiding principles are intended to design AI systems that prioritize human rights and inclusivity, ensuring equitable access to technologies regardless of socio-economic status or geographic location.

##### 3.1.4.3 Ethics guidelines for trustworthy artificial intelligence

The High-Level Expert Group on AI set up by the European Commission published in 2019 the *Ethics Guidelines for Trustworthy Artificial Intelligence*, that outlines a framework emphasizing lawfulness, ethics, and robustness in AI development (81). The guidelines identify principles such as respect for human autonomy, harm prevention, fairness and transparency, among other examples. These principles are operationalized through seven key requirements, including privacy governance and technical robustness, thereby offering a comprehensive approach to fostering ethical and trustworthy AI in the EU.

##### 3.1.4.4 EU AI act

The EU AI Act, published in the Official Journal of the EU on July 12, 2024, is the first comprehensive regulation on AI within the EU. It introduces harmonized rules for AI systems development and deployment (82). A risk-based classification system forms the central component of the Act, categorizing AI systems by (i) minimal risk, unregulated, (ii) high risk, that must undergo a conformity assessment, and (iii) unacceptable risk, which is prohibited. For high-risk systems, like the ones designed for healthcare purposes, the Act mandates these systems comply with strict regulations related to transparency, data governance, and human oversight. Regulatory sandboxes, structured and controlled environments for AI developers to develop, test and validate new AI systems in a supervised manner, are also being established across Member States to foster innovation while ensuring compliance with EU standards (83). A notable feature of this Act is the establishment of an AI Board, that acts as a central entity to ensure the successful implementation of the regulation across Member States (84).

The Board has the authority to invite relevant national and Union authorities, bodies, or experts to meetings and establish subgroups to address specific issues. A dedicated AI Board subgroup on public health could facilitate the exchange of best practices and coordination of national strategies in this field. This subgroup could play a key role in shaping governance policies and developing guidelines to help public health specialists navigate AI models and tools. Given the complexity of available studies and solutions, such a platform would enhance decision-making and preparedness for public health emergencies.

##### 3.1.4.5 European health data space

The EHDS regulation, published on 5 March 2025, is designed to establish a unified and secure framework for health data sharing across Member States (85). Its primary goal is to enhance individuals’ access and control over their personal electronic health data (primary use), while also enabling secondary uses, such as for research, policymaking, and preparedness and response to health threats, among other examples. In addition, this regulation seeks to streamline the internal market functioning by defining consistent legal and technical frameworks for the development, marketing, and use of electronic health record systems, ensuring alignment with EU values. Therefore, this regulation will set an important foundation that will contribute to the development of AI capabilities and foster AI-driven innovations in healthcare, by improving the health data quality and establishing procedures for the secondary use of health data.

##### 3.1.4.6 European ethical principles for digital health

Adopted in 2022, the European Ethical Principles for Digital Health provide a foundation for ethical and equitable digital health implementation in the EU (86). These principles emphasize humanistic values, citizen empowerment in managing health data, inclusivity, and environmental sustainability. A key focus is on ensuring AI systems are explainable, unbiased, and transparent, aligning with the EU’s commitment to trustworthy and inclusive innovation. These principles support the EHDS by guiding policymakers to prioritize citizen well-being and build trust in digital health solutions.

## 4 Key challenges and future recommendations

The integration of AI in public health surveillance holds great promise for enhancing precision, efficiency, and response times in managing health threats and medical countermeasures (87). However, several challenges must be addressed to harness AI’s potential, including ensuring data privacy, addressing biases (88, 89), enhancing transparency, and maintaining data quality and interoperability across systems. Furthermore, compliance with legal and ethical standards (90), building technical capacity, training the public health workforce, and fostering public trust are all critical components for the successful AI implementation in this field.

The EU has made significant progress toward responsible AI deployment, with the EU AI Act playing a pivotal role in setting standards for reliability and transparency in AI applications. However, the inherent complexity of selecting, using, and overseeing AI in the multifaceted public health ecosystem requires careful guidance. Policymakers and public health authorities must develop strategies and frameworks that foster responsible AI adoption, integrating principles that address governance, education, equity, ethics, and more.

A robust governance framework for data usage, sharing, and accountability must be established, with the EU AI board showing potential to play a crucial role in coordinating national strategies. This could be further supported by creating a subgroup dedicated to AI in public health. Continuous education and training programs should be implemented to support the public health workforce, encouraging the use of AI as a tool for professional development. Additionally, promoting inclusive policies and encouraging open-source AI are essential to strengthen equity in access to AI technologies.

Adherence to ethical and legal principles must be prioritized to ensure AI solutions comply with EU regulations and the European Ethical Principles for Digital Health. Regulatory sandboxes will be valuable for testing AI systems before deployment in public health contexts. Transparency and explainability of AI models should be prioritized, ensuring that decision-making processes and underlying algorithms are understandable to healthcare professionals, policymakers, and the public, while maintaining human oversight. Additionally, ensuring interoperability between AI and health systems is another critical element, addressing legal, organizational, semantic, and technical dimensions (91) to facilitate seamless integration and effective implementation of AI-driven solutions in public health.

Bias mitigation requires the systematic identification and correction of different types of confounding biases, such as selection, measurement and algorithmic biases, be it through regular bias audits, fairness-aware learning algorithms and the use of balanced, high-quality datasets, to uphold both equity and trust (92). Regular updates to AI systems that would be incorporating the latest clinical evidence, automated compliance checks and robust version control should preserve model performance, enhance explainability via tools or counterfactual explanations, and ensure alignment with evolving regulations. From a technological observation point, explainability should be candidly considered both in terms of how it can be achieved and what is beneficial from a development perspective, and how it can align with the point of view of the end-users and the target demographics. Here, the interpretation that can be achieved by patients and/or health professionals offers important cues on how the explainability of the AI is transmitted. Omitting explainability in clinical decision support systems undermines fundamental medical ethics and risks harmful outcomes for both individual patients and public health. Finally, data privacy and cybersecurity must be embedded from end to end—using privacy-preserving techniques such as strong encryption, granular access controls and continuous threat monitoring—to safeguard sensitive health information and maintain system integrity.

Cross-border data sharing is crucial for public health as communicable diseases have no borders. While the European Health Data Space (EHDS) Regulation (85) lays important groundwork to enable health data sharing, cross-border data integration is still a challenge. For example, issues regarding data standardization, quality and consistency increases the complexity of integrating these heterogeneous datasets, requiring complex data engineering and sophisticated analytics to ensure compatibility and meaningful insights. Additional, when integrating global health data, it is necessary to comply with data protection laws of multiple jurisdictions which are not harmonized globally. Non-compliance can lead to legal consequences or loss of public trust. There are some international agreements in place, such as the International Health Regulations (IHR) (93), which facilitate global data sharing for health surveillance. However, not all countries are parties to these agreements, and even within those that are, the specifics of data-sharing may vary, creating a barrier to seamless integration. To overcome these barriers, a concerted global effort is needed, combining technological innovation with strong legal frameworks and international collaboration. Standardizing data formats, enhancing data security, fostering interoperability, and aligning legal and ethical standards across countries will be key to enabling effective global public health surveillance (94).

Collaboration among stakeholders is essential to promote inclusive, diverse AI solutions and encourage responsible AI-human collaboration in decision-making. A holistic approach to AI adoption will also require the development of public health policies that support the ecological sustainability of AI technologies. Additionally, benchmarking frameworks can help assess the quality of health data and the AI systems performance; while monitoring and evaluation frameworks should be established to assess the responsible use of AI.

In low-income countries (LICs), the application of AI faces significant challenges, primarily due to insufficient digital infrastructure, a lack of accurate digitized data, and a limited pool of skilled talent in AI-related fields. Additionally, LICs often struggle with weak institutional frameworks and the absence of robust regulations governing data privacy and safety, all of which hinder their ability to fully capitalize on the transformative potential of AI. These barriers exacerbate existing global inequalities, preventing LICs from accessing the full benefits of AI-driven innovation. However, opportunities exist to bridge these gaps and accelerate AI adoption in LICs. By leveraging the absorptive capacity framework, LICs can focus on strengthening institutional structures, investing in human capital through targeted educational programs, and designing tailored policies that address their specific socio-economic contexts. Moreover, fostering collaborative networks with advanced economies and facilitating technology transfer can accelerate knowledge exchange, allowing LICs to adapt global AI technologies to local needs. These strategies can help LICs overcome barriers to AI integration, foster sustainable development, and enable these countries to fully participate in the global AI landscape (95).

The successful implementation of these recommendations requires an interconnected, multi-faceted approach to AI adoption. By addressing these challenges comprehensively, AI can reach its full potential to transform public health surveillance globally, benefiting both individuals and communities, while ensuring that AI technologies are deployed in a responsible, ethical, and impactful manner.
