# Supplementary material for: Harnessing artificial intelligence for enhanced public health surveillance: a narrative review
Source: Front Public Health. 2025 Jul 30;13:1601151. doi: 10.3389/fpubh.2025.1601151 (PMC12343694; doi:10.3389/fpubh.2025.1601151)
Supplement: Supplementary file 1 [file Table_1.DOCX]

**Supplementary Table 1.** Summary of selected AI use cases, categorized into studies focusing on single AI models (section A) and multiple AI models (section B), with potential applications in public health, particularly for identifying and monitoring public health threats.

| Entry | AI Model | Type | Study Application | Data Sources | Target Pathogen / Use Case | Potential for Public Health | Ref. |
| --- | --- | --- | --- | --- | --- | --- | --- |
| Section A – Single AI Models | | | | | | | |
| 1 | GSRNN | **Deep Learning** | Forecasting of regional influenza trends with spatial correlation | CDC data from the FlueView platform, that compiles information from 100 public and 300 private laboratories in the USA | Influenza | Localised outbreak prediction, optimises surveillance and resource allocation by modelling inter-regional transmission | 21 |
| 2 | Metapopulation model with machine-learned anonymized mobility map | **Machine Learning** | Retrospective forecasting of influenza outbreaks using mobility data | Google Aggregated Mobility Research Dataset, CDC FluView, EpiQuery NYC syndromic surveillance, flu test data, demographics, from New Jersy (USA) and Australia | Influenza | Disease modelling and forecasting, with particular effectiveness in regions with strong surveillance systems;  High-quality mobility data is highlighted for disease modeling and forecasting, particularly in regions with detailed surveillance systems | 26 |
| 3 | RF | **Machine Learning** | Dengue transmission risk prediction and spatial risk mapping | Epidemiological, demographic and environmental past dengue exposure data from 2006 to 2013 from Singapore | Dengue | Accurate spatial prediction of high-risk areas, supporting prioritization of vector control efforts | 28 |
| 4 | LSTM | **Deep learning** | Outbreak focal points prediction, enabling the prediction of a potential peak within 2 weeks, and estimating the potential outbreak’s ending point around June 2020 | Data sets of confirmed COVID-19 cases, number of fatalities and recovered patients by the end of each day available until March 31, 2020, from Johns Hopkins University and Canadian Health authority data sources | COVID-19 | Public health forecasting, enabling timely intervention and resource allocation | 37 |
| 5 | LSTM | **Deep Learning** | Evolution and spread analysis of COVID-19 throughout the country of Bangladesh | Daily confirmed cases, recovered cases, and death cases of COVID-19 from March 2020 to August 2021 extracted from Health Division of the Government of the Republic of Bangladesh | COVID-19 | Disease monitoring, enabling peak and focal outbreaks identification to support management decision-making | 38 |
| 6 | DELPHI | **Machine Learning and AI augmented SEIR framework** | Predict, with moderate accuracy, the effects of underreporting cases and the impact of governmental intervention measures on the outbreak progression, while also identifying potential future outbreak hotspots | Reported cases up until April 2020 | COVID-19 | Disease outbreak spots prediction, enabling the identification of ideal locations for clinical trials due to focal outbreaks and community spread | 41 |
| 7 | INFEKTA | **Agent-based modelling based on SEIR** | Represented Bogotá as a network of homes, workplaces, schools, markets, and public transport stations, where agents interact daily based on predefined probabilities for infection and recovery | Multiple information based on public infrastructure in Bogotá, namely public transports, schools, demographics | COVID-19 | Agent-based modelling to support decision-making in infectious disease management by accurately assessing how infection spreads based on spatiotemporal parameters | 46 |
| 8 | ABM/ODE model | **Agent-based modelling incorporating SEIR** | Evaluate the effects of different lockdown scenarios and event characteristics on the transmission of diseases | Urban districts from Germany with population of 100000 | COVID-19 | Effectiveness analysis of preventive measures applied to pandemics | 47 |
| 9 | ChatGPT-4 | **Large Language Model** | Assist disease modelling and other models through an AI assistant | No defined datasets | N/A | Disease modelling democratization, offering a scalable solution adaptable to diverse needs across various geographies, languages, and populations | 48 |
| 10 | Supervised classification algorithm to classify the tweets (K-means), followed by unsupervised clustering algorithm (logistic regression classification) and a multistep qualitative analysis | **Machine Learning** | Examine the varying discourse amongst pro- and anti-vaccine populations | Twitter (now X) posts collected from Oct 2019 to Nov 2019 (daily monitoring for tweets); academic literature, popular news articles and twitter were used to identify and select the keywords | Vaccination public opinion | Impact analysis of positive and negative discourse on vaccination and population engagement | 68 |
| 11 | SIRVD-DL | **Deep Learning combined with infectious disease mathematical models** | Prediction of COVID-19 trends including infections, recoveries, and deaths | Johns Hopkins University System Science and Engineering Center (global cumulative confirmed cases, cumulative cured cases, and cumulative deaths, etc); Our World website data (confirmed cases, deaths, hospitalizations, testing, vaccinations, etc.) | COVID-19 | Predictive model for infection rate, recovery, and death rate based on confirmed cases, with potential for both modelling and forecasting applications | 29 |
| Section B – Multiple AI Models | | | | | | | |
| 12 | CNN and RNN | **Deep Learning** | Influenza epidemic prediction via time-series forecasting | Influenza surveillance data from Japan and the USA | Influenza | Accurate epidemic evolution forecasting, allowing faster response measures and better disease surveillance | 20 |
| 13 | CNN and RNN | **Deep learning** | Viral mutations forecasting, assessing virulence, adaptability, and spillover risk of zoonotic pathogens | Zoonotic reservoirs, human-animal interfaces, and environmental factors; publicly available genome databases (GenBank and GISAID) | Emerging pathogens with zoonotic / pandemic potential | Early detection and surveillance of virus evolution, enabling proactive pandemic preparedness by identifying high-risk mutations and spillover events | 30 |
| 14 | BioBERT and BERN2 (GIX Framework) | **Large Language Model** | Automated extraction of gene and protein interactions from scientific literature | Scientific publications (PubMed abstracts) | General use for outbreak-related genetic data; not pathogen-specific | Efficient processing of scientific biomedical literature to speed the identification of therapeutic options in potential outbreaks, with significant potential to expedite outbreak response research | 31 |
| 15 | DNN, LSTM and ARIMA | **Machine learning and Statistical modelling** | Disease prediction using internet and environmental data | Search queries, social media data, temperature, humidity collected between 1 January 2016 to 29 July 2017 from Korea. | Chickenpox, Scarlet Fever, Malaria | Early disease progress identification, allowing the identification of spreading patterns | 24 |
| 16 | LSTM, SVM and ARIMA | **Machine learning and statistical modelling** | Monthly incidence and case numbers of Hepatitis E forecasting | Shandong Center for Disease Control and Prevention data collected between 2005 and 2017 from China. | Hepatitis E | Monthly disease incidence tracking and cases number monitoring, supporting timely intervention and resource allocation | 25 |
| 17 | SVM, GBM and RF | **Machine learning** | Global distribution prediction of *Aedes aegypti* and *Aed*es *albopictus* mosquitoes | Multidisciplinary datasets – including occurrence records, social factors, and meteorological factors | Vector-borne diseases (Zika, Dengue, Chikungunya) | Risk area identification for mosquito-borne diseases for public health planning and disease control strategies; | 27 |
| 18 | ARIMA, SARIMA and Prophet | **Machine learning and statistical modelling** | New daily cases, and cumulative reported cases prediction (even asymptomatic that contribute to disease spreading) | Dataset published by the WHO of new and cumulative confirmed cases of COVID-19 between May 1, 2020, and November 30, 2021 | COVID-19 | Disease surveillance and monitoring, supporting case identification and decision-making | 40 |
| 19 | SVR and ANN | **Machine Learning** | R_0_ of multiple diseases prediction | Unclear dataset | Multiple diseases | R₀ prediction for new, unseen networks, demonstrating their generalizability, allowing diseases to be modeled and studied in a more efficient way | 43 |
| 20 | ARNN-LNE, ARNN vs SVR and SVM | **Machine Learning** | Early warning signals identification of a disease outbreak before a critical transition from a normal state to an outbreak state | Datasets of COVID-19 confirmed cases from six nations or regions, including Germany, Canada, Italy, Netherlands, Spain, and parts of Europe between 2020 and 2021 | COVID-19 | Early identification of outbreak states and early disease outbreak warning | 39 |
| 21 | RF, RF-UFA and ARIMA vs GLM and GAM | **Machine Learning and linear classifications** | New dengue cases and early disease outbreaks detection | Weekly dengue cases and meteorological data from three cities (Iquitos of Peru, San Juan of Puerto Rico, and Singapore) spanning various time frames, such as 1990 to 2010 | Dengue | Early identification of disease outbreaks and focal hotspots, supporting decisions on actions to control the spread before it achieves community spreading; identifying disease vectors | 42 |
| 22 | Convolutional sequence-to-sequence neural networks, based on the STConvS2S architecture vs VAR, ARMA and SIRD | **Machine learning** | COVID-19 spread and transmission modelling | Reported cases between 1 March 2020 and 28 February 2021 | COVID-19 | Accurate prediction of the natural course of disease progression and expansion throughout the community | 44 |
| 23 | MLP and ANFIS vs SEIR and SIR | **Deep learning and Machine learning** | COVID-19 outbreak modelling | Data from worldometers between 18 January and 22 March 2020 in Italy, Germany, Iran, USA and China | COVID-19 | Long-term prediction of COVID-19 disease progression | 45 |
| 24 | EIOS | **Natural Language Processing and Machine Learning and BERT** | Enhancement of traditional disease surveillance systems | Open-source data from online news outlets, blogs, forums, and social media platforms, scanning thousands of articles and posts each day from multiple languages. to train the main model. Example: Tokyo Olympics, FIFA World Cup 2022 and multiple country COVID-19 data | COVID-19 and/or multiple different types of public health issues ranging from gastrointestinal disorders, to foodborne, waterborne or zoonosis | Patterns and trends identification indicative of emerging health threats. It can be used in large scale world events to give decision makers the ability to understand and predict certain risks.  Real-time monitoring, where it categorizes health-related events and geolocates potential outbreak | 50-53 |
| 25 | BlueDot | **Natural Language Processing and Machine Learning** | Disease monitoring and focal outbreaks detection through predictive datasets | Data collected from hundreds of thousands of sources, including available information from official public health organizations, digital media and reports, global airline ticketing data and travel logs, livestock and disease vectors health reports and population demographics | Multiple disease monitoring | Identification of potential community disease spreading from both identified and unidentified diseases – including hotspots, cold spots and spatial outliers, predicting potential destinations most likely to experience outbreaks. Regular alerts are sent to clients, providing actionable insights to mitigate risks and informing public health responses | 55-60 |
| 26 | EpiWatch | **Machine Learning (prioritization algorithms, natural language processing) and other AI technologies (e.g., custom LLMs developed based on openAI proprietary API and ChatGPT-4)** | Automate data collection and analysis, allowing it to identify signals of emerging epidemics quickly and efficiently than traditional surveillance methods | Wide range of sources, including media reports, press releases, official government reports, and social media from multiple languages.  Per use depending on the study at hand, for example, Mumps during 2016-2019 | Multiple potential diseases | Multiple seasonal diseases monitoring, including tools such as FluCast (influenza monitoring), Epirisk (epidemiological risk stratification tool), and ORIGINS (tracing epi/pandemic root origin) | 61-64 |
| 27 | SENTINEL (Synergistic process of DNN, CNN and LSTM) vs SVM | **Machine Learning** | Disease surveillance and trends identifiable in social media | Twitter (now X) and CDC disease reports | Social media monitorization and outbreak identification | Symptom report spikes detection, and generation of real-time situational awareness for public health monitoring; real-time processing of public social medical data | 66 |
| 28 | GPT-3.5, GPT-4, Mistral 7B, and Mixtral 8x7B | **Large Language Model** | Extraction of public stances towards vaccination from social media posts and gauge public opinion and misinformation | 1000 English-language vaccination-related post from Twitter (now X), extracted from 2019-2022 | Vaccines and public opinion or misinformation campaigns aimed at them | Population perception identification and monitoring regarding public health interventions, such as vaccinations, to adjust risk communication and community engagement activities and prevent the spread of misinformation | 67 |
| 29 | Llama-2-3, Mistral, and Flan-T5 base models, GPT-4 | **Large Language Model** | Public health tasks, focusing on classification across three areas (burden, risk factors, and interventions) and extraction from free text (academic, news, social media, and questionnaires) | Manually annotate seven datasets with public health specific annotations using a combination of internal, synthetic, and external free text sources. | Identifying disease spread, contact tracing, contact spread and epidemiological factors | Extraction of insights from social-media platforms, enhancing surveillance, research and intervention strategies | 69 |

Abbreviations:

ABM – Agent-Based Modelling; AI – Artificial Intelligence; ANN – Artificial Neural Network; ANFIS - Adaptive Neuro Fuzzy Inference System; ARIMA – AutoRegressive Integrated Moving Average; ARMA – Autoregressive moving-average model; CNN – Convoluted Neural Network; CDC – Centers for Disease Control and Prevention; DNN – Deep Neural Network; GIX Framework – Gene Interaction Extraction Framework; GAM – Generalized Additive Model; GLM – Generalized Linear Model; GSRNN – Graph-based Spatio-Temporal Recurrent Neural Network; LLM – Large Language Model; LSTM - Long Short-Term Memory; ML – Machine Learning; MLP - Multi-Layered Perceptron; N/A – Not Applicable; ODE - Ordinary differential equation; RF – Random Forest; RF-UFA – Random Forest-Unweighted Frequency Analysis; RNN – Recurrent Neural Network; SARIMA – Seasonal AutoRegressive Integrated Moving Average; SEIR – Susceptible, Infected, Exposed, Recovered; SIRD – Susceptible, Infected, Recovered, Deceased; SVM – Support Vector Machine; SVR – Support Vector Regression; VAR – Vector Autoregression.
